# Does Eating Addiction Favor a More Varied Diet or Contribute to Obesity?—The Case of Polish Adults

**DOI:** 10.3390/nu12051304

**Published:** 2020-05-02

**Authors:** Marzena Jezewska-Zychowicz, Aleksandra Małachowska, Marta Plichta

**Affiliations:** Institute of Human Nutrition Sciences, Warsaw University of Life Sciences (SGGW-WULS), 159C Nowoursynowska Street, 02-787 Warsaw, Poland; marzena_jezewska_zychowicz@sggw.edu.pl (M.J.-Z.); marta_plichta@sggw.edu.pl (M.P.)

**Keywords:** overweight, obesity, food addiction, eating addiction, food intake variety, eating behavior, overeating

## Abstract

The rapidly increasing prevalence of overweight and obesity indicates a need to search for their main causes. Addictive-like eating and associated eating patterns might result in overconsumption, leading to weight gain. The aim of the study was to identify the main determinants of food intake variety (FIV) within eating addiction (EA), other lifestyle components, and sociodemographic characteristics. The data for the study were collected from a sample of 898 Polish adults through a cross-sectional survey in 2019. The questionnaire used in the study included Food Intake Variety Questionnaire (FIVeQ), Eating Preoccupation Scale (EPS), and questions regarding lifestyle and sociodemographic factors. High eating addiction was found in more than half of the people with obesity (54.2%). In the study sample, physical activity at leisure time explained FIV in the greatest manner, followed by the EPS factor: eating to provide pleasure and mood improvement. In the group of people with obesity, the score for this EPS factor was the best predictor of FIV, in that a higher score was conducive to a greater variety of food intake. Sociodemographic characteristics differentiated FIV only within groups with normal body weight (age) and with overweight (education). In conclusion, food intake variety (FIV) was associated with physical activity at leisure time, and then with EPS factor “Eating to provide pleasure and mood improvement”, whereas sociodemographic characteristics were predictors of FIV only within groups identified by body mass index (BMI). Nevertheless, our observations regarding the eating to provide pleasure and mood improvement factor and its associations with food intake variety indicate a need for further research in this area. Future studies should also use other tools to explicitly explain this correlation.

## 1. Introduction

In spite of the growing prevalence of overweight and obesity, determining their main risk factors is still a challenge. Body weight and body mass index (BMI) are greatly influenced by energy intake and its adequacy [1]. However, the link between diet and those anthropometric parameters cannot be solely assessed on the basis of calorie intake, but should also include other elements of dietary patterns (eating frequency, diet quality, food variety, or proportions between different food groups) [2]. Lifestyle-related factors, such as unhealthy dietary patterns but also low physical activity, inadequate sleep hygiene, poor stress management, and tobacco smoking, can majorly alter energy intake and expenditure, and thus induce a positive energy balance [3]. Research shows that lifestyle factors are correlated with each other. Low physical activity is associated with the consumption of unhealthy foods [4,5]. In turn, less stress or negative as well as highly positive effects are associated with engagement in healthy behaviors, especially in physical activity [6]. Physical activity can reduce stress as well as negative emotions and, at the same time, enhance positive emotions. By contrast, human emotional functioning is associated with food, including emotional eating [7]. Physically active emotional eaters may want to eat when under emotional distress; however, they also choose more healthy foods to cope with this distress [8]. These interrelationships between selected lifestyle components, but also within human psychological functioning, implicate the necessity of including such parameters while exploring eating behaviors characteristics.

Some dietary patterns, such as uncontrolled excessive consumption, may resemble addictive behavior, and some foods may have addictive potential [9]. Gearhardt et al. [10] developed the first tool to assess FA, the Yale Food Addiction Scale (YFAS), as well as the follow-up, YFAS 2.0 [11]. These tools enable identification of addictive-like eating behaviors particularly towards highly processed and palatable foods. Elevated YFAS and YFAS 2.0 scores are both positively associated with body mass index (BMI), binge eating symptoms, and weight-cycling [11]. Research suggests that people diagnosed as food addicts consume more calories [12,13,14,15,16], especially derived from processed, energy-dense foods like confectionary, fast-food, and salty snacks, and their diet is higher in fat [12,14,16] than non-food-addicted individuals. Several studies have revealed that food addiction can be correlated with lower consumption of fruit, vegetables, and other core products [13,15].

Overeating might be associated with one of the following eating styles: restrained, emotional, or external. In restrained eating, when someone is following a strict dietary regimen, eating something forbidden may induce an “all-or-nothing” reaction leading to overconsumption [17]. Negative, positive, or neutral emotional states (e.g., sadness, anxiety, joy, boredom) might also increase food intake (emotional eating). Lastly, environmental factors, such as availability of food or presence of others eating, might also affect the consumption in so-called external eating [18]. Studies have found that emotional eating might favor undesirable food behaviors, including higher intake of snacks [19,20], “fast-food” [19], and sweet foods [21,22], whereas external eating may increase total calorie intake [19] as well as predispose to higher consumption of snacks [19,23]. Although dietary restraint can be conducive to lower intake of sweets [19] and total energy intake [19,24,25], it may simultaneously serve as a risk factor for excessive body weight [19,24,25]. The possible explanation of this phenomenon might be related to the possibility that people following strict dietary rules may be more susceptible to external and emotional eating, which can lead to weight gain [26]. Food-related thoughts are believed to be another crucial factor in the etiology of excessive food consumption as they can induce a specific food craving. When the urge to fulfill this craving arises, it can be difficult to resist overeating. Food preoccupation might therefore take the form of obsession [27].

In previous studies, also those using YFAS or YFAS 2.0, dietary assessment did not take into account food intake variety (FIV), which reflects the number of food products consumed by the individual. For many years, FIV was being promoted as a vital component of dietary guidelines. It was believed that a wider range of products will improve intake of macro- and micronutrients and provide adequate nutritional status [28]. Although a systematic review of 26 studies has shown that it is still unclear how total FIV affects body weight and measures of body adiposity [28], this parameter is of special concern to medical scientists and health professionals due to the growing obesity epidemic [29]. Results from the studies assessing the relationship between FIV and diet quality or eating habits remain inconsistent. Some research suggests a negative impact [30,31], whereas several studies have found that FIV might favor healthy eating habits, such as adequate intake of fruit and vegetables [32,33], or predispose to greater diet quality [34,35]. The existing research results suggest that sociodemographic characteristics, such as gender and age, can differentiate assessed variables and their correlations [30,33,34].

We assume that differences in food intake variety (FIV) can be explained by eating addiction assessed using the Eating Preoccupation Scale (EPS). However, we hypothesize that EPS explains the differences in FIV to a lesser extent than some components of lifestyle (i.e., physical activity, following a diet, smoking) but the importance of these factors may vary depending on BMI. Thus, the aim of the study is to assess eating addiction in a group of Polish adults, and to then answer the following questions: (1) Does eating addiction show a relationship with food intake variety? (2) Do such lifestyle components as following a diet, smoking, and physical activity differentiate the food intake variety more than the eating addiction? (3) Do the relationships between the examined variables differ after taking BMI into account?

## 2. Materials and Methods

### 2.1. Study Design and Sample Collection

The data were collected from February to March 2019 through a cross-sectional quantitative survey. The study was approved by the Ethics Committee of the Faculty of Human Nutrition and Consumer Science, Warsaw University of Life Sciences, in Poland on the 29 October 2018 (Resolution No. 22/2018). Informed consent to participate in the study was collected from participants.

According to the study design, recruitment and data collection were conducted by a research agency—ARC Market and Opinion. Adults aged 18–65 were recruited from the panel (epanel.pl) of approximately 64,000 adults. After sending an invitation to participate in the study, 2025 people gave their consent to participate in the study. Quota selection using gender, age, place of residence, and education was used to ensure the representativeness of the Polish population. During the recruitment, 78 people stopped filling out the questionnaire during the interview, and 932 people did not qualify due to filling the quota, while eight people were removed from the database at the collection control stage because of errors indicating the lack of credibility of their answers. As a result, the study consisted of 1007 participants. The computer-assisted web interviewing (CAWI) technique was used to collect all data. During the data check, 71 participants were excluded from the sample due to missing data, i.e., body mass and height, which did not allow calculation of the BMI. Then, during the data analysis, one more criterion of exclusion was used, namely being underweight (body mass index (BMI) < 18.5 kg/m^2^). Thirty-eight participants were excluded from the analyses due to BMI lower than 18.5 kg/m^2^. The total sample consisted of 898 people.

### 2.2. Food Intake Variety

Food intake variety was assessed using the food consumption frequency method, applying Food Intake Variety Questionnaire (FIVeQ) [36]. Information on the consumption of 63 food product groups over the last 7 days was collected using the FIVeQ questionnaire [36]. Quantity was specified for each product: seven slices for cereal products, seven cups for dairy and beverages with the exception of wine (quantity defined as 1 glass of wine—100 mL) and spirits (one shot of liquor—50 mL), amount sufficient for one slice of bread well covered (approx. 20 g) for cold cuts and sausages, 10 cubes for chocolate, and two tablespoons for the rest of the food products (e.g., groats, nuts, fish, and butter). The participant declared the consumption of such quantity of each product within the last 7 days (Yes/No). Food intake variety is expressed in the food intake variety index (FIVeI). FIVeI was calculated as the number of product groups eaten weekly (maximum 60 products/week) after excluding 3 groups of alcoholic beverages (beer, wine, vodka, and other strong alcohols). According to the methodology and assessment criteria developed by the authors of the questionnaire [35], the following four groups of people with a varied food intake (FIV) were distinguished:

Inadequate FIV (<20 food products weekly)
Sufficient FIV (20–29 food products weekly)
Good FIV (30–39 food products weekly)
Very good FIV (≥40 food products weekly)

### 2.3. Eating Addiction

Eating Preoccupation Scale (EPS) was used to assess eating addiction [37]. EPS consists of 18 statements, to which the respondent answers on a scale of 1—hardly/never; 2—rarely; 3—sometimes; 4—often; up to 5—almost/always (Table 1). This scale allows measuring an overall score of eating addiction and three EPS factors, which include focusing on eating activities; eating to provide pleasure and mood improvement; and compulsion to eat and loss of control over food. The overall score (range from 18 to 90 points), which was the sum of all ratings, allows evaluating a person’s behavioral characteristics for eating addiction (EA) included in EPS. A score above 48 points indicates a high EA, 40–48 points an average EA, and below 40 points a low EA [37].

The internal compliance of the questionnaire was assessed using Cronbach’s coefficient, which was 0.89. Internal stability, measured using a correlation coefficient in studies conducted after 6 weeks on a group of 30 women, was 0.72. Validity of the Eating Preoccupation Scale was tested by assessing the correlation of its results with the results of the Eating Related Behaviors Questionnaire [37], which measures the tendency toward habitual and emotional overeating, but also following dietary restrictions.

### 2.4. Physical Activity and Other Lifestyle Factors

Self-reported physical activity was recorded in the questionnaire on a 3-point scale: 1—”low”, 2—”moderate”, and 3—”high” [38]. The description of the scale was presented separately for physical activity during leisure and work/school time. For leisure time, “low” was described as “sedentary lifestyle, watching TV, reading the press, books, light housework, taking a walk for 1–2 h a week”; “moderate”—”walks, cycling, gymnastics, gardening or other light physical activity performed for 2–3 h a week “, and “high”—”cycling, running, working on a plot or garden, and other sports activities requiring physical effort, taking up more than 3 h a week”. “Low” activity at work/school time was described as “over 70% of the time in a sitting position”, “moderate” as “approximately 50% of the time in a sitting position and about 50% of time moving”, and “high” as “about 70% of the time in motion or doing physical work associated with a lot of effort” [38].

Two questions were used to assess smoking: “*Do you smoke cigarettes?*” (Yes/No) and “*If you smoke, how many cigarettes a day do you smoke?*” (I smoke occasionally; up to 10 a day, 10–20 a day, more than 20 a day). In addition, respondents answered the question “*Have you followed a special diet in the last 3 months?*” (Yes/No).

### 2.5. Sociodemographic Characteristics

The questionnaire collected information about sociodemographic characteristics of the study sample, i.e., gender, age, education, and place of residence. Body mass index (BMI) was calculated using self-reported body weight and height and categorized according to International Obesity Task Force (IOTF) standards [39]. During the data analysis, three categories of respondents were identified, i.e., people with normal weight (BMI between 18.5 and 24.99 kg/m^2^), overweight (BMI between 25.0 and 29.99 kg/m^2^), and obesity (BMI ≥ 30 kg/m^2^).

### 2.6. Statistical Analysis

Descriptive statistics were performed. The chi-square test and the one-way analysis of variance ANOVA test were used to compare variables, and *p* < 0.05 was considered significant.

The classification tree was used to determine independent variables explaining differences in food intake variety. This method was used because it allows computing both numerical and categorical data. Moreover, it offers clear graphic data presentation and is easy to interpret [40]. Separate classification trees were made in the study sample, and then in a group of people with normal body weight, overweight, and obesity. The method CHAID (chi-squared automatic interaction detector) was used to build the tree. The first node (node 0) is always the distribution of the dependent variable (FIV). The next nodes can include sociodemographic variables (gender, age, education, place of residence), variables describing eating addiction (eating addiction—overall score, three factors of eating addiction: focusing on eating activities, eating to provide pleasure and mood improvement, compulsion to eat and loss of control over food) and lifestyle variables (following a diet, smoking, physical activity during leisure time, and work/school time).

Statistical analysis was conducted using IBM SPSS Statistics for Windows, version 24.0 (IBM Corp, Armonk, NY, USA).

## 3. Results

### 3.1. Characteristics of the Study Sample

The sample consisted of 898 participants (433 women and 465 men) aged 18 to 65 years. Some details concerning sociodemographic characteristics of the study sample are displayed in Table 1.

More men than women were overweight or obese. Among people with normal weight the majority were people of the age of 18–34, while among overweight and people with obesity respondents aged 45–65 were the most numerous in this group. The average age of people with overweight and obesity did not differ, but was significantly higher compared to people with normal body weight. Education and place of residence did not differentiate groups identified according to BMI (Table 2).

### 3.2. Food Intake Variety and Other Lifestyle Factors

About 60% of the study sample displayed good or very good food intake variety (36.8% and 23.7%, respectively). FIV did not differ in BMI groups (Table 3).

Slightly more than 10% of participants declared following a diet. Almost two-thirds of the study participants (64.0%) declared they did not smoke. In the study sample, there were less heavy smokers (10 or more cigarettes a day) than light smokers (16.6% and 19.4%, respectively). About two-fifths of the study sample (38.3%) described their physical activity at work/school as low, and the same numbers of people evaluated their leisure activities in the same way. More than one-half of people with BMI ≥ 30 kg/m^2^ (57.6%) declared low physical activity in leisure time. More people with overweight than ones with normal body weight indicated low activity in leisure time (37.7% and 32.8%, respectively) (Table 3).

### 3.3. Eating Addiction

Over two-fifths of study sample (42.1%) displayed a high eating addiction (EA) on the EPS. The mean value of the overall score from the EPS was 46.4 points, which indicates the average EA. Only differences in the overall score of EPS between people with normal weight and people with obesity were shown. The mean value of the overall score in the obese group exceeded 48 points and, therefore, meant a high EA. Low EA was displayed by 33.7% of people with normal body weight and by almost three times less of those with obesity (13.1%). However, a high EA was found in more than half of the people with obesity (54.2%) and in more than one-third of people with normal body weight (37.7%). Compulsive eating and loss of control of food consumption characterized eating behaviors of people with obesity to a higher extent compared to people with normal body weight. There were differences in the mean score for the “Focusing on eating activities” factor in the BMI groups. The larger the BMI, the more people were focused on eating behaviors were (Table 4).

### 3.4. Relationship between Food Intake Variety and Eating Addiction

**Figure 1:**
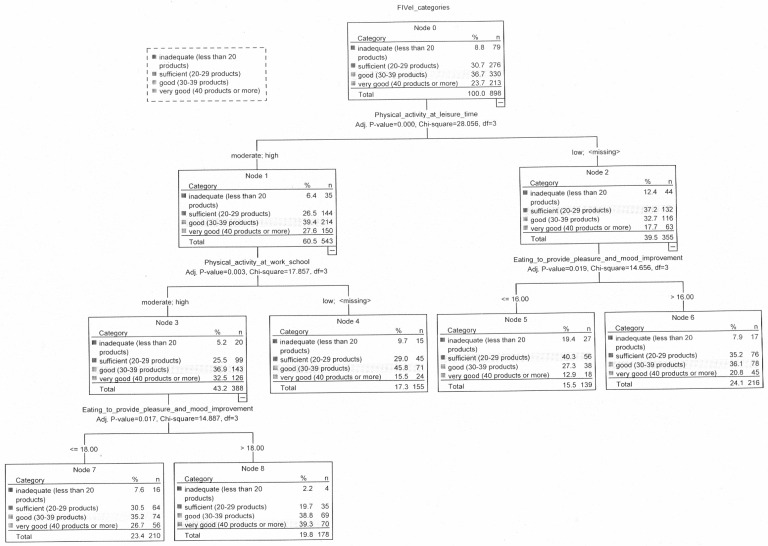
Relationship between food intake variety, eating addiction, selected lifestyles variables and sociodemographic characteristics in the study sample.

Food intake variety (FIV) has shown differences only due to EPS factor “Eating to provide pleasure and mood improvement” (Figure 1). In the group of people with high or moderate physical activity at leisure time and at work/school time, a higher score for the EPS factor “Eating to provide pleasure and mood improvement” (above 18 points) favored an increase in FIV (nodes 7 and 8). Almost two-fifths of people with a score above 18 had very good FIV. Similarly, in the group of people with low physical activity at leisure time (nodes 5 and 6), a higher score for this EPS factor (above 16 points) was conducive to a greater variety of food intake (Figure 1).

**Figure 2:**
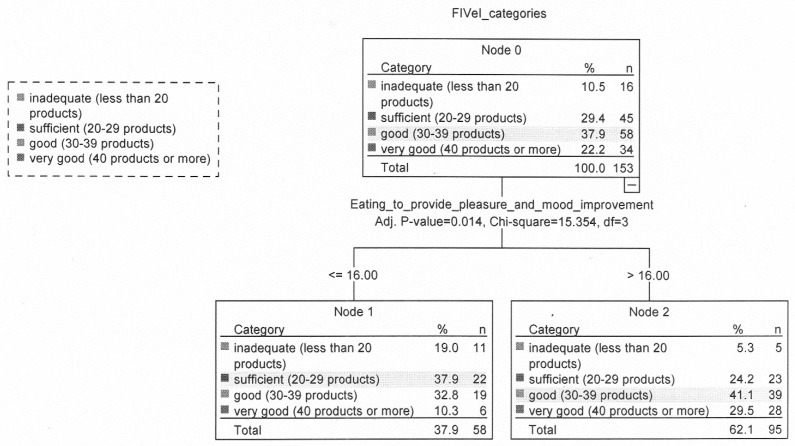
Relationship between food intake variety, eating addiction, selected lifestyles variables and sociodemographic characteristics in the group with obesity.

In the group of people with obesity, the score of EPS factor “Eating to provide pleasure and mood improvement” was the most powerful predictor for FIV (nodes 1 and 2). A higher score for this EPS factor (above 16 points) was conducive to a greater variety of food intake. Almost three times more people with a score above 16 (29.5%) than with a score of 16 and below (10.3%) had a very good FIV (Figure 2).

### 3.5. Relationship between Food Intake Variety and Lifestyle and Sociodemographic Variables

In the study group, FIV has shown differences due to physical activity at leisure time (nodes 1 and 2) and physical activity at work/school (nodes 3 and 4), —as seen in Figure 1. Higher FIV was demonstrated in people with moderate and high physical activity at leisure time (*p* < 0.001). Over one-quarter of people (27.6%) with moderate or high physical activity and 17.7% of those with low physical activity at leisure time were characterized by very good FIV. Twice as many people with low physical activity in their leisure time were characterized by inadequate FIV compared to other people. Twice as many people with high and moderate physical activity in leisure time and the same physical activity at work/school showed very good FIV (32.5%) compared to people with low physical activity at work/school (15.5%) (nodes 3 and 4) (Figure 1).

**Figure 3:**
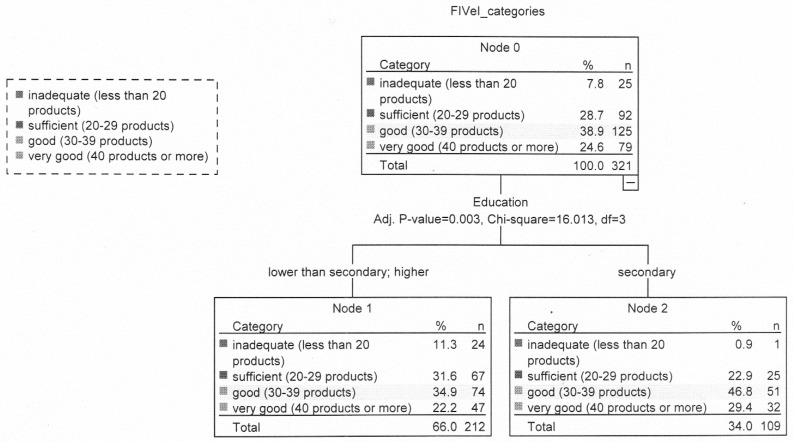
Relationship between food intake variety, eating addiction, selected lifestyles variables and sociodemographic characteristics in the group with overweight.

In the group of people with overweight (nodes 1 and 2), more people with secondary education than the other categories had good FIV (46.8%, 34.9%, respectively) and very good FIV (29.4%, 22.2%, respectively (Figure 3).

**Figure 4:**
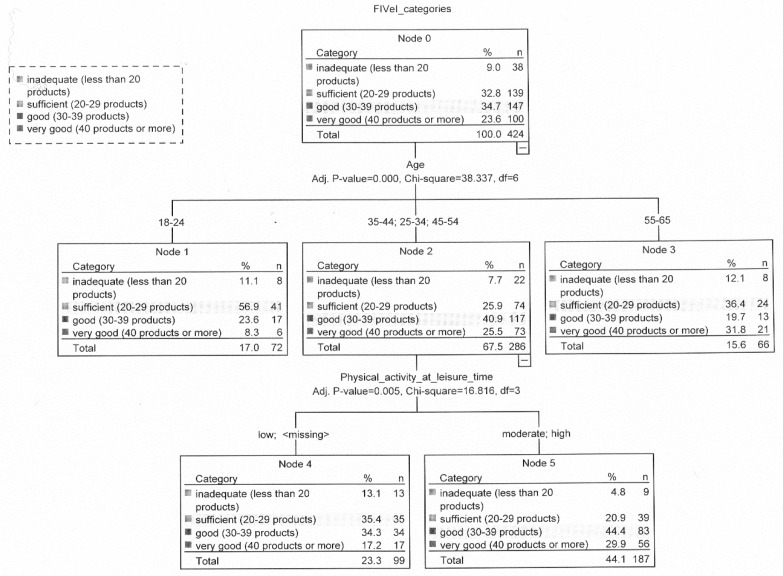
Relationship between food intake variety, eating addiction, selected lifestyles variables, and sociodemographic characteristics in the group with normal body weight.

In people with normal body weight, FIV differed among age groups (nodes 1, 2, and 3). The number of people aged 18–24 was the least when it came to showing very good FIV (8.3%), while most people aged 55–65 (31.8%). More than two-thirds of people aged 18–24 had inadequate FIV (11.1%) or sufficient FIV (56.9%). By contrast, more than three-quarters of people aged 25–54 years were characterized by good (40.9%) or very good FIV (25.5%). In this age group (nodes 4 and 5) more people with moderate or high physical activity in leisure time than others had good (44.4%, 34.3%, respectively) and very good (29.9%, 17.2%, respectively) FIV (Figure 4).

## 4. Discussion

The study provided some support for our hypotheses that differences in food intake variety (FIV) can be explained by eating addiction assessed using the Eating Preoccupation Scale (EPS). The results concerning associations between FIV and EPS indicate that only one of the EPS factors, “Eating to provide pleasure and mood improvement”, was related to FIV. Moreover, this indicator did not serve as the most important predictor of FIV, as physical activity during leisure time explained this parameter in a greater manner. Other EPS factors and total EPS did not differentiate FIV. In view of the previous research, it can be assumed that “Eating to provide pleasure and mood improvement” as a factor correlated with FIV might favor both overall dietary variety and dietary variety within particular food groups only. Few available studies, which attempted to explain differences in food intake variety, indicate that this parameter might be linked to the amount of food consumed with regard to selected foods [13,41]. People with overeating tendencies usually opt for a wide range of food products, yet it only applies to products considered as palatable. Thus, a wide range of palatable foods might be a factor involved in the development of addictive-like eating behaviors [42]. Other authors also point out that not all foods seem to be equally related to addictive-like eating behaviors. Foods rich in refined carbohydrates and added fat are more likely to be consumed in an addictive manner than low-processed foods [43,44]. High-fat and high-sugar foods were consumed more frequently among individuals who met the criteria of the Yale Food Addiction Scale for food addiction [13]. These foods also appear to trigger behavioral responses that are consistent with addictive-like eating behavior, for example such foods are frequently consumed during binge episodes [45]. Moreover, foods high in fat and sugar are more likely to be intensely craved [41,46,47] and consumed in greater quantities in response to negative affect [48,49]. The results of these studies are consistent with those obtained in our research among people with obesity. Within this group, the EPS factor “Eating to provide pleasure and mood improvement” was the most important factor in explaining FIV. Almost three times more people with a higher score of this EPS factor were characterized by higher FIV in comparison to people scoring lower on this subscale. It might be considered as a cause of overconsumption though longitudinal research is required to determine the direction of causality.

Our hypothesis was also supported, that EPS explains the differences in FIV to a lesser extent than some components of lifestyle (i.e., physical activity, following a diet, smoking) but the importance of these factors may vary depending on BMI. Physical activity at leisure time was the most important predictor of FIV in the study sample, while in the groups distinguished by BMI, differences in FIV predictors were observed. Greater food intake variety (FIV) correlated with moderate or high physical activity during leisure time, which may be the result of higher awareness of healthy lifestyle, healthier food choices, and greater adherence to dietary rules among physically active people [50,51]. Similarly as for the whole study group, the association between food intake variety (FIV) and physical activity in leisure time was supported among individuals with normal body weight aged 25–54, and greater FIV was observed in those more physically active within this age group. According to our best knowledge, an association between FIV and physical activity has not been the subject of previous research. Nevertheless, some studies have shown that physical activity favors healthier food choices among adults [52,53,54]. However, a few studies have revealed that being physically active might not always determine healthy eating nor prevent unfavorable eating behaviors [55]. On the premise that FIV might be linked to both health benefits and the higher intake of unhealthy foods, our results indicating that greater FIV is observed among physically active people is supported by previous studies.

Higher levels of physical activity observed in individuals presenting eating addiction symptoms might be caused by an attempt to make up for the excessive amount of calories consumed through exercise [56]. Physically active people are able to self-regulate food intake more precisely due to the effect of working out in lowering the reactivity of the brain reward system to food stimuli [57]. Moreover, among individuals in the group of moderate or high physical activity, both in leisure or school/work time, a positive correlation was seen for the result of “Eating to provide pleasure and mood improvement” subscale and FIV, which can be linked to self-contentment associated with satisfaction from living a healthy lifestyle, beneficial for health and wellbeing. A similar correlation was noted in the group with low physical activity in leisure time. It seems that low physical activity can induce food cravings in a manner resembling an addiction mechanism [58]. In those individuals, food can serve as a major source of pleasure, since sedentary behaviors favor food consumption. This association was not seen for the eating addiction total score in our study, and these results are supported by Li et al. [56].

Sociodemographic features as predictors of FIV were noticed only in groups separated due to BMI. Food intake variety in individuals with normal body weight was associated with age, which can be confirmed by previous research. Due to involutional processes along with environmental and psychological factors, older people tend to change their eating habits, which leads to lower calorie as well as macro- and micronutrient intake. Inadequate intake of nutrients increases the risk of malnutrition [59,60]. Greater FIV among older adults in our study, including having the largest number of very good FIV and, at the same time, the largest number of inadequate FIV, indicates that the recommendations on dietary diversity in older people [35,60] are being partly fulfilled. Higher FIV in older people was also noted by Drewnowski et al. [61].

The age was not associated with food intake variety in individuals with overweight and obesity. However, people with overweight with secondary education had greater FIV than the others. The impact of education on FIV in people with overweight may be explained in different ways. Environment, awareness of physical activity, dietary knowledge, and health literacy as well as social roles and cultural norms related to health and nutrition seem to be significant factors affecting this correlation [62]. Among people with lower educational status, a less varied diet might be linked to their living environment with limited access to more diverse and affordable fresh foods, but also to other components of a healthy lifestyle, including safe places for physical activity. On the other hand, alcohol, tobacco, and fast-food might be more accessible, which are conducive to a high-calorie diet combined with sedentary behaviors [63]. By contrast, people with higher education are expected to have more opportunities for being physically active, but also greater access to diverse food products. Educational status might be considerably associated with salary, and thus influence food choices and food variety [64,65]. Moreover, higher educated people should be more predisposed to favoring new or unfamiliar foods [66]. Nonetheless, the above possible explanations and mechanisms involving dietary knowledge and health literacy [67,68] cannot explain the results revealed in our study indicating that among those in the overweight group, people with higher education had lower FIV than individuals with secondary education. Despite having greater nutrition knowledge, more highly educated individuals might conform more to cultural norms, e.g., the thin ideal, which is often perceived as a condition of success [69]. Some authors suggested that body weight dissatisfaction might serve as a driver for unhealthy dieting behaviors [70,71]. It can be assumed that in our study sample, overweight people with higher education could have been particularly susceptible to social norms which, in turn, led to following a strict dietary regimen, thus resulting in lower FIV [70,71].

### Strengths and Limitations

The strength of our study is its relatively large sample, representative of the Polish population in terms of the region of residence, gender, education, and age. Although our findings are specific to the Polish population and should not be generalized to populations of other cultural backgrounds, the observations could be of potential use in designing research and interventions. The analysis of relationships between eating addiction and lifestyle elements of great importance for health, i.e., diet (dietary variety and following a diet), and physical activity brought a wider perspective on adequate diet. To the best of our knowledge, this paper is the first to study the association between eating addiction and food intake variety. The use of the hitherto unknown Eating Preoccupation Scale can be considered as both a strength and a weakness. On the one hand, it may be noticed by other researchers and recognized as a tool that deserves further use. On the other hand, the use of this scale is a limitation of our study. The measure of eating addiction used in the present study (EPS) has not been extensively used and there is a need for additional research on its psychometric properties and its association with measures of related constructs such as food addiction, emotional eating, and binge eating. Additionally, this cross-sectional study design does not provide an opportunity to find a causal relationship between food intake variety and other variables. Some limitations are related to the potential biases that may occur when self-reported data are analyzed [72]. People tend to underreport their weight and overreport their body height [73], which may have led to underestimation of individuals with excessive body weight according to the BMI categories in our research. Self-reported indicators of lifestyle can be considered not quite satisfying, however, and using other measurement indicators confirms the results from the analysis of self-reported data [74].

## 5. Conclusions

The study found that food intake variety (FIV) was associated with physical activity at leisure time, and then with the EPS factor “Eating to provide pleasure and mood improvement”, whereas sociodemographic characteristics were predictors of FIV only within groups determined by BMI. In the study sample, physical activity both at leisure and at work/school time proved to be a stronger predictor than the EPS factor related to pleasure and mood. However, the EPS factor “Eating to provide pleasure and mood improvement” was the only predictor of FIV among people with obesity. Sociodemographic characteristics differentiated FIV only within the group with normal body weight (age) and with overweight (education). Based on the findings of this study, it is possible to better understand the relationships between food intake variety and some components of lifestyle, including addictive behaviors. Moreover, additional focus on the groups identified by BMI and the performed analysis will allow the results to be used in dietary practice. However, there is still a need for further research involving the use of tools that can identify the “eating addiction” construct. Symptoms of eating addiction might serve as a marker of disordered eating, while early diagnosis can significantly affect both prevention and treatment of overweight and obesity. Further research attempting to clarify the association between FIV and EA should use also other tools to explicitly explain this correlation.

## Figures and Tables

**Table 1:** The Eating Preoccupation Scale (EPS).

Statements from the Eating Preoccupation Scale (EPS)	Mean Score ± Standard Deviation *
EPS factor: Focusing on eating activities	
2. I think about eating and about my body weight	3.0 ± 1.2
6. I believe that my relationship with food is terrible	2.3 ± 1.1
8. I feel embarrassed about the amount of food I eat	2.2 ± 1.1
9. I plan ahead for situations when I will be able to eat alone	1.9 ± 1.0
10. I am worried about being unable to control the amount of food consumed	2.3 ± 1.1
16. I have a low self-esteem because of my uncontrolled eating	2.1 ± 1.1
EPS factor: Eating to provide pleasure and mood improvement	
1. Eating is a very important part of my life	3.4 ± 1.1
11. Eating greatly enhances my mood	3.2 ± 1.0
12. Eating is a great pleasure of mine	3.6 ± 1.0
13. I make myself “food feasts” for no clear reason	2.2 ± 1.1
17. I feel great satisfaction after an abundant meal	2.8 ± 1.1
18. I am willing to sacrifice other pleasures for eating	2.3 ± 1.0
EPS factor: Compulsion to eat and loss of control over food	
3. I eat vast amounts of high-calorie foods in a short period of time	2. 6 ± 1.0
4. I snack throughout the day	2.9± 1.0
5. I eat even when I am not feeling hunger	2.4 ± 1.0
7. I eat more than I had planned	2.7 ± 1.0
14. I wake up to eat at night	1.8 ± 1.0
15. I clear up my plate even when I am not feeling hungry anymore	2.9 ± 1.2

* 5-point scale: 1—hardly/never; 2—rarely, 3—sometimes, 4—often, 5—almost/always.

**Table 2:** Characteristics of the study sample.

Variables		Total (N = 898)		18.5 kg/m^2^ ≤ BMI < 25 kg/m^2^ (N = 424)		25.0 kg/m^2^ ≤ BMI < 30 kg/m^2^ (N = 321)		BMI ≥ 30 kg/m^2^ (N = 153)	
		N	%	N	%	N	%	N	%
Gender *	Female	433	48.2	234	55.2	131	40.8	68	44.4
	Male	465	51.8	190	44.8	190	59.2	85	55.6
Education	Lower than secondary	348	38.8	153	36.1	123	38.3	72	47.1
	Secondary	309	34.4	153	36.1	109	34.0	47	30.7
	Higher	241	26.8	118	27.8	89	27.7	34	22.2
Place of residence	Rural area	329	36.6	159	37.5	113	35.2	57	37.3
	City ≤ 100,000 residents	291	32.4	140	33.0	106	33.0	45	29.4
	City > 100,000 residents	278	31.0	125	29.5	102	31.8	51	33.3
Age *	18–24 years	97	10.8	72	17.0	18	5.6	7	4.6
	25–34 years	205	22.8	117	27.6	62	19.3	26	17.0
	35–44 years	209	23.3	105	24.8	67	20.9	37	24.2
	45–54 years	168	18.7	64	15.1	70	21.8	34	22.2
	55–65 years	219	24.4	66	15.5	104	32.4	49	32.0
Age (years)	Mean; standard deviation	42.0; 13.7		38.0 ^a^; 13.3		45.6 ^b^; 13.1		45.5 ^b^; 12.9	
Height (cm)	Mean; standard deviation	171.4; 9.5		170.8 ^a^; 9.1		172.4 ^a^; 9.6		170.8 ^a^; 9.9	
Weight (kg)	Mean; standard deviation	76.6; 15.8		65.7 ^a^; 9.2		81.0 ^b^; 10.2		97.6 ^c^; 13.8	
BMI (kg/m^2^)	Mean; standard deviation	26.0; 4.5		22.4 ^a^; 1.8		27.2 ^b^; 1.4		33.4 ^c^; 3.6	

N—number of participants; * Significant at *p* < 0.001 between BMI groups (chi-square test); ^a,b,c^ Different letters in each line indicate significant differences at *p* < 0.05 between BMI groups (ANOVA test).

**Table 3:** Food intake variety and other lifestyle characteristics of the study sample.

Variables		Total Sample (N = 898)		18.5 kg/m^2^≤ BMI < 25 kg/m^2^ (N = 424)		25.0 kg/m^2^≤ BMI < 30 kg/m^2^ (N = 321)		BMI ≥ 30 kg/m^2^ (N = 153)	
		N	%	N	%	N	%	N	%
Food intake variety—FIV	inadequate	79	8.8	38	9.0	25	7.8	16	10.5
	sufficient	276	30.7	139	32.7	92	28.7	45	29.4
	good	330	36.8	147	34.7	125	38.9	58	37.9
	very good	213	23.7	100	23.6	79	24.6	34	22.2
Following a diet	yes	97	10.9	42	10.0	37	11.6	18	12.0
Number of cigarettes smoked **	no smoking	575	64.0	266	62.7	223	69.5	86	56.2
	less than 10 cigarettes a day	174	19.4	91	21.5	56	17.4	27	17.6
	10 or more cigarettes a day	149	16.6	67	15.8	42	13.1	40	26.2
Physical activity during work/school time	low	329	38.3	137	34.3	123	39.4	69	47.3
	moderate	329	38.3	158	39.5	120	38.5	51	34.9
	high	200	23.4	105	26.2	69	22.1	26	17.8
Physical activity during leisure time ***	low	344	38.8	137	32.8	120	37.7	87	57.6
	moderate	415	46.8	208	49.8	153	48.1	54	35.8
	high	128	14.4	73	17.4	45	14.2	10	6.6
Food intake variety—FIV (number of products)	Mean; standard deviation	32.6; 10.7		32.4 ^a^; 10.6		33.0 ^a^; 10.5		32.6 ^a^; 11.3	

N—number of participants; ** Significant at *p* < 0.01; *** Significant at *p* < 0.001 between BMI groups (chi-square test). ^a^ Having the same letter means no significant differences at *p* < 0.05 between groups (ANOVA test).

**Table 4:** Eating addiction in the study sample.

Variables		Total Sample (N = 898)		18.5 kg/m^2^ ≤ BMI < 25 kg/m^2^ (N = 424)		25.0 kg/m^2^ ≤ BMI < 30 kg/m^2^ (N = 321)		BMI ≥ 30 kg/m^2^ (N = 153)	
		N	%	N	%	N	%	N	%
Eating Preoccupation Scale (EPS)—total score ***	low	237	26.4	143	33.7	74	23.1	20	13.1
	average	283	31.5	121	28.6	112	34.8	50	32.7
	high	378	42.1	160	37.7	135	42.1	83	54.2
Eating Preoccupation Scale (EPS)—total score	Mean; standard deviation	46.4; 11.0		45.2 ^a^; 11.6		46.7 ^a,b^; 10.6		49.1 ^b^; 9.9	
EPS factor: Focus on eating activities	Mean; standard deviation	13.7; 4.8		12.9 ^a^; 5.0		13.8 ^b^; 4.6		15.5 ^c^; 4.1	
EPS factor: Eating to provide pleasure and mood improvement	Mean; standard deviation	17.5; 4.4		17.5 ^a^; 4.5		17.6 ^a^; 4.2		17.5 ^a^; 4.3	
EPS factor: Compulsion to eat and loss of control over food	Mean; standard deviation	15.2; 4.2		14.8 ^a^; 4.4		15.3 ^a,b^; 4.1		16.1 ^b^; 3.9	

N—number of participants; *** Significant at *p* < 0.001 between BMI groups (chi-square test). ^a,b,c^ Different letters in each line mean significant differences at *p* < 0.05 between groups (ANOVA test).
